# WHERE CARE FALLS SHORT: MAPPING THE SUPPLY–DEMAND GAP IN ADRD CARE ACROSS AN AGING UNITED STATES

**DOI:** 10.1093/geroni/igaf122.4048

**Published:** 2025-12-31

**Authors:** Yung Chun, Oejin Shin, Soobin Park, Sojung Park

**Affiliations:** Washington University in St. Louis, St. Louis, Missouri, United States; Illinois State University, Normal, Illinois, United States; Washington University in St. Louis, St. Louis, Missouri, United States; Washington University in St. Louis, St. Louis, Missouri, United States

## Abstract

The growing prevalence of Alzheimer’s disease and related dementias (ADRD) poses urgent challenges for equitable access to long-term care across the United States. While demand for ADRD care is driven by an aging population and rising cognitive health needs, the availability of facilities and specialized services remains uneven, raising concerns about geographic disparities. This study integrates multiple data sources to evaluate the spatial balance of ADRD demand and care infrastructure at the county level. Demand is estimated using American Community Survey data on older adults with cognitive difficulties, while supply is drawn from National Care Planning Council records covering nine facility types across 31 states. Explanatory Spatial Data Analysis (ESDA) is applied to map demand, supply, and their balance, with results further disaggregated by facility type. Preliminary findings reveal sharp disparities: demand is concentrated in large metropolitan counties, the Sunbelt, and parts of the Midwest and Appalachia. Facilities cluster along the East Coast, California, Florida, and selected Midwestern metros, while deficits are greatest in the rural South, Great Plains, and Mountain West. Specialized services such as adult day programs and Alzheimer’s centers are heavily urban, whereas generalized services—nursing homes, assisted living, and retirement facilities—are more dispersed but still unevenly distributed. These findings highlight a persistent spatial mismatch between population needs and care infrastructure. The concentration of specialized services in metropolitan centers risks leaving rural and micropolitan communities underserved. Addressing these inequities is critical to promoting equitable aging, reducing caregiver burdens, and ensuring timely access to ADRD care.

